# Supplementary material for: Serum Visfatin/NAMPT as a Potential Risk Predictor for Malignancy of Adrenal Tumors
Source: J Clin Med. 2022 Sep 22;11(19):5563. doi: 10.3390/jcm11195563 (PMC9572558; doi:10.3390/jcm11195563)
Supplement: Supplementary file 1 [file jcm-11-05563-s001.zip › jcm-1882332-supplementary.pdf]

Table S1. Sample size analysis for comparison of visfatin serum levels between patients with adrenocortical carcinomas and benign adrenocortical tumors.

		Type I Error - Alpha			
		0.20	0.10	0.05	0.01
Type II Error - Beta	0.20	11 + 13	15 + 17	**19 + 22**	28 + 33
	0.10	16 + 18	21 + 24	**25 + 29**	35 + 41
	0.05	20 + 23	25 + 29	31 + 36	42 + 49
	0.01	30 + 35	36 + 42	42 + 49	55 + 65

In bold type minimal sample sizes for 80-90% statistical power assuming significance level α=0.05. The first element describes the cases and the second the controls.

Table S2. Sample size analysis for ROC analysis with visfatin serum level as a predictor discriminating between patients with adrenocortical carcinomas and benign adrenocortical tumors.

		Type I Error - Alpha			
		0.20	0.10	0.05	0.01
Type II Error - Beta	0.20	11 + 14	16 + 19	**20 + 24**	30 + 36
	0.10	16 + 19	21 + 25	**26 + 31**	37 + 44
	0.05	20 + 24	26 + 31	31 + 37	44 + 53
	0.01	30 + 36	37 + 44	43 + 51	57 + 68

In bold type minimal sample sizes for 80-90% statistical power assuming significance level α=0.05. The first element describes the cases and the second the controls.

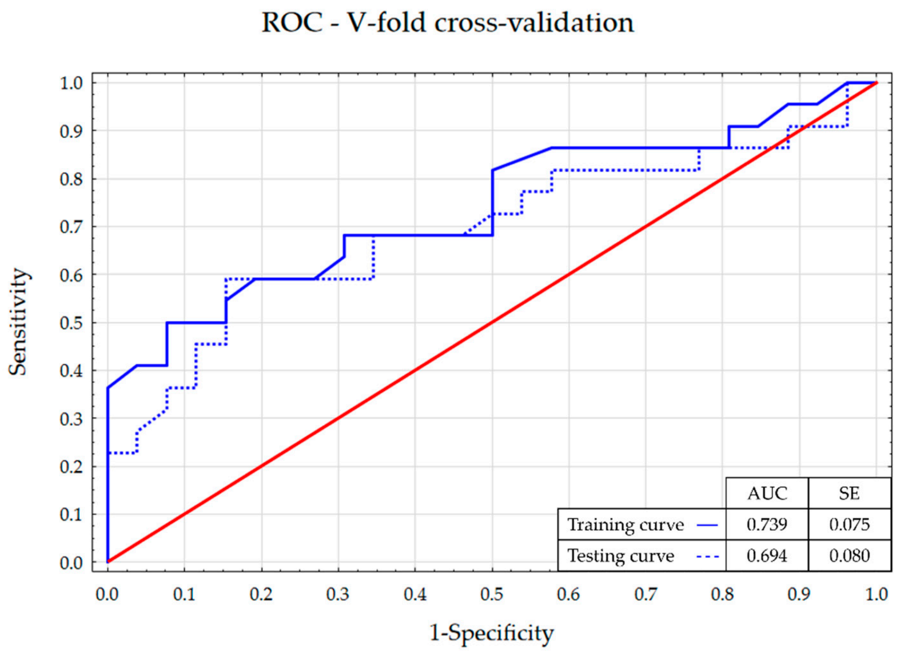

Figure S1. V-fold cross-validation—learning ROC curves for the univariate logistic regression model including visfatin serum concentration (AUC, area under curve; SE, standard error).
